# Nature of Host Immunity during Hepatitis B Virus Infection and designing Immune Therapy

**DOI:** 10.5005/jp-journals-10018-1256

**Published:** 2018-05-01

**Authors:** Sheikh MF Akbar, Mamun Al-Mahtab, Sakirul I Khan

**Affiliations:** 1 Department of Medical Sciences, Toshiba General Hospital, Tokyo, Japan and Miyakawa Memorial Research Foundation, Tokyo Japan; 2 Department of Hepatology, Bangabandhu Sheikh Mujib Medical University, Dhaka, Bangladesh; 3 Department of Anatomy and Embryology, Graduate School of Medicine, Ehime University, Ehime, Japan

**Keywords:** Chronic hepatitis B, Immune therapy, Pathogenic immunity, Protective immunity, Therapeutic immunity.

## Abstract

Hepatitis B virus (HBV) infections represent one of the major public health problems in global context. More than 2 billion people in the world have been infected with this virus at some point of time in their life and millions are chronically infected, indicating that chronic HBV-infected subjects remain as a living source of HBV transmission. The public health impact of this is tremendous. Considerable numbers of chronic HBV-infected individuals would eventually develop progressive liver diseases and their complications like hepatic failure, liver cirrhosis (LC), and hepatocellular carcinoma (HCC). Epidemiological studies have suggested that about 0.6 to 1.2 million people die annually from HBV-related liver diseases. These figures about death due to HBV and sufferings from HBV-related diseases indicate a notion of medical emergencies about HBV. In addition to these, the impact of HBV on health care delivery system moves beyond these numbers of HBV-related patients and HB-related deaths. This is because significant insights have already been developed about epidemiology, virology, and pathogenesis of HBV. Also, an effective and widely used preventive vaccine is available against HBV. In addition to these, antiviral drugs against HBV have been developed from early 1980s and several such drugs are now available commercially in the open market around the worldwide. Unfortunately, the ongoing therapeutic regimens could not stand the test of time and new insights about HBV pathogenesis are required for the development of new, novel, and evidence-based therapies for chronic HBV infections.

**How to cite this article:** Akbar SMF, Al-Mahtab M, Khan SI. Nature of Host Immunity during Hepatitis B Virus Infection and designing Immune Therapy. Euroasian J Hepato-Gastroenterol 2018;8(1):42-46.

## DIVERSITIES OF NATURAL COURSE OF HBV INFECTION

The natural course of HBV infection differs among individuals and seems to be dependent on several factors, some of which may be related to virus and the others may be host-related. Almost all estimates indicate that about 2 billion people have been infected by HBV at some point of time in their life; however, about 80 to 90% (~1.7 billion) control both HBV replication and liver damages by some mechanisms and those are not yet completely clear and understandable.^1^ About 240 to 370 million HBV-infected individuals show persistent HBV replication, a fact that is evaluated by presence of hepatitis B surface antigen (HBsAg) in the blood. Also, HBV deoxyribonucleic acid (DNA) would be present in their blood. About 20% patients with chronic HBV infection exhibit evidences of liver damages and some of them develop complications like liver failure, LC, and HCC.^1-3^ Thus, it remains a challenging issue as to why the same virus causes highly diverse pathological patterns. Definitely, if the factors related to these can be revealed, new and novel therapeutic approaches may be developed for containment of adverse effects of HBV infection.

## HBV-INDUCED LIVER DAMAGES: POSSIBLE MECHANISMS

Hepatitis B virus is a noncytopathic virus, and its direct role on liver damage has not been elucidated in animal models or in patients with chronic hepatitis B (CHB) or other HBV-infected subjects. To assess the liver damaging capacity of HBV, several lines of HBV transgenic mouse (HBV TM) containing different levels of HBV DNA, HBsAg, and hepatitis B e-antigen (HBeAg) have been developed. However, none of this HBV TM developed evidences of liver damage and progressive liver diseases, such as LC and HCC.^4-6^

In human, the levels of HBV DNA, HBsAg, or HBeAg as well as HBeAg-positivity or HBeAg-negativity and HBsAg-positivity or HBsAg-negativity do not correlate with the extent of liver damages in CHB patients.^7-9^ Patients with CHB with very high levels of HBV DNA and HBeAg may be found among the group of inactive HBV carriers. On the contrary, low levels of HBV DNA and patients expressing anti-HBe may be found in the group of patients with progressive liver diseases. Thus, the direct role of HBV in inducing and sustaining liver damages and hepatic complications could not be substantiated yet in animal model of HBV or in patients with chronic HBV infection.

In this scenario, it remains to be clarified how liver damages are induced and maintained in chronic HBV-infected individuals. Also, it is important to discuss why the extents of liver damages are so diverse among patients to patients. In fact, most of these queries could not be properly explained due to narrow animal range of chronic HBV infection. And, most relevant information could not be retrieved from CHB, LC, and HCC patients due to ethical and scientific limitations.

Since the levels of HBV DNA or magnitude of HBV-related antigens do not correlate with liver damage in CHB patients, most investigators have focused on host factors in addition to viral factors to get insights about these important issues. Circumstantial and experimental data suggest that the immune responses of the host act like a double-edge sword in chronic HBV infection. On the one hand, the host immunity is mainly responsible for containment of HBV replication and control of liver damages. This is a natural response of the vertebrate to invading pathogens. On the other hand, this observation is supported by the fact that acquisition of chronicity after HBV infection is much less in adults than in neonates as the adult immune system is mature compared with those of new born and neonates.

## “PATHOGENIC IMMUNITY” IN CHRONIC HBV INFECTION

The study of Ando et al^10^ showed a temporal relationship between host immunity and hepatocyte damage in an animal model of HBV infection, HBV TM, a mice model that is prepared by microinjecting the HBV genome in fertilized eggs of mice of different strains. Accumulation of nonspecific mononuclear cell infiltrates was observed in the damaged liver of HBV TM that expressed HBV DNA and HBV-related antigens. Similar observations were also found in patients with CHB. Although several mononuclear infiltrates were detected in the liver of CHB patients, the levels of nonantigen-specific lymphocytes were higher in the liver of CHB patients experiencing liver damage and failing to control HBV replications compared with the patients controlling HBV replication and containing liver damages.^11^ The proportion of natural killer (NK) cells to total immunocytes is normally high in healthy liver; however, the biological significance of this phenomenon remains mostly unknown. In this context, it has been found that the number of NK cells in the liver of CHB patients is further elevated and these are responsible for inducing liver damages.^12^ Dunn et al^13^ investigated the mechanisms underlying NK-induced liver damage in CHB patients and found that the majority of NK cells infiltrating damaged liver were activated by NK cells. Additionally, the concentration of activated NK cells was significantly higher among CHB patients with high alanine transaminase (ALT) levels compared with patients with low ALT levels.^14^ Also, the mechanisms underlying localization of NK cells have been elucidated to certain extents. HBV-infected liver secretes interferon (IFN)-gamma-induced chemokines, including CXCL9 and CXCL10, which may influence the recruitment of nonantigen-specific immunocytes including NK cells.^15-17^ The recruitment of such nonantigen-specific immunocytes can cause chronic inflammation that may induce liver damage and chronic hepatitis. Tumor necrosis factor (TNF)-related apoptosis-inducing ligand (TRAIL) is a primary ligand involved in the NK-mediated destruction of hepatocytes. The NK cells from control subjects express low levels of TRAIL; however, expression is upregulated in NK cells from CHB patients. The receptor for TRAIL is also upregulated on the surface of hepatocytes in CHB patients^18^; thus, NK cells expressing TRAIL may induce apoptosis of liver cells expressing the ligand. In addition to a TRAIL-dependent mechanism of apoptosis, the Fas pathway has also been implicated in causing lysis of hepatocytes by NK cells in CHB patients.^15,18^ In addition to NK cell-induced mediators, multiple cytokines may be involved in the pathogenesis of CHB and also for inducing liver damages. Cytokines, like interleukin (IL)-8, IFN-alpha, and IL-1-beta, show increased expression in CHB patients compared with those of healthy controls.^19^ The expression levels of IL-6, TNF-alpha, transforming growth factor (TGF)-beta, hepatocyte growth factor, and epidermal growth factor are also elevated in CHB patients. Moreover, IL-22 has been shown to increase the proinflammatory activity of TNF-alpha.^20^ Thus, persistent infection with HBV induces a cascade of immunological changes that allow accumulation of nonantigen-specific immune modulators either via cytokine or chemokine or NK pathways. These have been implicated in inducing and progressing liver damages in CHB.

Persistent inflammation in CHB patients activates hepatic stellate cells, myofibroblasts, and fibroblasts, which initiate collagen, laminin, fibronectin, and proteoglycan production and deposition: These factors may lead to the pathogenesis of liver fibrosis and LC. The activation of these cells is regulated by proinflammatory cytokines, such as TGF-p, IL-6, TNF-a, and CCL-21, among other stimuli.^21^ Moreover, LC may be an outcome of the collective dysregulation of liver regeneration processes.

## PROTECTIVE IMMUNITY IN CHRONIC HBV INFECTION

High levels of antigen-specific immunocytes have been detected in the liver of CHB patients who control HBV replication and contain liver damage. However, direct implications of this correlation are still elusive and their roles during recovery from liver damages are still lacking.^11^ Several investigators have assessed the safety and therapeutic potential of HBV antigen-specific immune modulation in CHB patients. Pol et al^22^ have shown that administration of HBsAg in CHB patients was safe and effective in reducing the concentrations of HBV DNA and containing the extents of liver damage. Subsequent several clinical trials also showed that HBV antigen-specific immunity were safe and were capable of reducing HBV DNA and liver damages.^23-27^ These factors clearly indicate that HBV antigen-specific immune modulation is not of pathogenic type.

In addition to HBsAg, a combination of HBsAg and anti-HBs was administered to Chinese CHB patients by Wen et al.^23,28^ A combination of antiviral drugs and therapeutic vaccines has also been used to induce antigen-specific immunity in CHB patients and were found to limit HBV replication and control liver damage.^29^ An hepatitis B core antigen (HBcAg) epitope-based vaccine has also been deemed safe and effective in CHB patients,^30^ and recently, a phase I/II clinical trial using an antigen-specific immune modulator containing HBsAg and HBcAg has started. The HBsAg/HBcAg-based vaccine is safe in CHB patients in whom it reduced HBV DNA in 50% of individuals. Normalization of ALT was also observed in all recipients of HBsAg/HBcAg-based therapeutic vaccine.^31^

## DESIGNING IMMUNE THERAPY FOR CHB PATIENTS BASED ON THE UNDERSTANDING ABOUT PATHOGENIC IMMUNITY AND PROTECTIVE IMMUNITY

Elucidation of the dichotomous nature of immunity in CHB is increasing and more and more information have been piling up regarding the roles of these immunities in CHB patients. A temporal relationship between the pathogenic effects of nonantigen-specific immunity and the protective effects of HBV antigen-specific immunity has been observed, but more works would be required to have a consensus about this highly diverse subject. Inducible HBV antigen-specific immunity has not been shown to have adverse effects in CHB patients and is nonpathogenic; however, long-term assessments of such patients with a particular focus on autoimmune phenomena have not been performed. Although inducible nonantigen-specific immunity seems to be pathogenic in nature, nucleoside analog and IFN have also shown protective and therapeutic effects in limited numbers of CHB patients.^32-35^ These observations raise several important questions regarding the immunity of CHB patients. It is an open query if there is any conversion of nonantigen-specific to antigen-specific immunity in CHB patients. Also, there is a need to have broad understanding about cytotoxic T lymphocytes (CTLs) in CHB patients. Cytotoxic T lymphocytes have been denoted as a population of immunocytes that kill their target cells; however, HBcAg-specific and HBsAg-specific CTLs do not kill hepatocytes. Rather, they seem to play a protective role in containing liver damage and HBV replication. Although the mechanisms underlying these activities are unknown, antigen-specific CTLs may produce cytokines that may regulate down grade of viral replication in a noncytopathic manner. It is possible that the observed effects are due to a combination of traditional CTLs with cytotoxic properties and CD8 T+ cells with protective properties. Taken these facts, it seems that evidence-based immune-therapeutic approaches may be formulated by techniques that would allow induction and maintenance of HBV antigen-specific immunity in CHB patients and regulate the adverse effects of nonantigen-specific immunity. However, phases I, II, and III clinical trials of these immune-therapeutic agents should be accomplished with an aim to elucidate mechanism of their actions.
